# Supplementary material for: Demographic factors associated with within-individual variability of lung function for adults with cystic fibrosis: A UK registry study
Source: J Cyst Fibros. 2024 Sep;23(5):936–42. doi: 10.1016/j.jcf.2024.05.013 (PMC11409769; doi:10.1016/j.jcf.2024.05.013)
Supplement: Supplementary Data S1 — Supplementary Raw Research Data. This is open data under the CC BY license http://creativecommons.org/licenses/by/4.0/ [file mmc1.pdf]

Supplementary material for the article

"Demographic factors associated with within-individual variability of lung function for adults with cystic fibrosis: a UK registry study"

A Statistical model

The final specification of the model is as follows. The mean submodel is

$$Y_{ij} = \beta_0^\mu + \beta_1^\mu \text{F508Homozygous}_i + \beta_2^\mu \text{DiagnosisAfterFirstYear}_i + f_1^\mu(\text{Age}_{ij}, \text{Sex}_i) + f_2^\mu(\text{YearofBirth}_i) + \upsilon_i + \varepsilon_{ij}$$

$$i = 1, \dots N; \quad j = 1, \dots, n_i \quad (1)$$

The variability submodel is as follows($\varepsilon_{ij} \sim N(0, \sigma^2_{\varepsilon_{ij}})$):

$$\sigma_{\varepsilon_{ij}} = \exp\left(\beta_0^\sigma + \beta_1^\sigma \text{F508Homozygous}_i + \beta_2^\sigma \text{DiagnosisAfterFirstYear}_i + f_1^\sigma(\text{Age}_{ij}, \text{Sex}_i) + f_2^\sigma(\text{YearofBirth}_i) + \omega_i\right) \quad (2)$$

Distribution of the random effects:

$$\begin{pmatrix} \upsilon_i \\ \omega_i \end{pmatrix} \sim N\left[\begin{pmatrix} 0 \\ 0 \end{pmatrix}, \begin{bmatrix} \sigma^2_\upsilon & \rho\sigma_\upsilon\sigma_\omega \\ \rho\sigma_\upsilon\sigma_\omega & \sigma^2_\omega \end{bmatrix}\right]. \quad (3)$$

The standard mixed model with random intercept is a particular case of MELSM when $\sigma^2_{\varepsilon_{ij}}$ is constant for all individuals at all measurements. In that case, υ_i is the only random effect in the model, accounting for the individual departure from the population average.

The use of random effects makes the interpretation of MELSM not dissimilar from the one in standard mixed models. In the mean submodel, the estimated coefficients will quantify the association between the covariates and the lung function, keeping constant the other variables. The random effect in the mean submodel will inform about additional shifts to the individual's FEV_1 with respect to other individuals with the same covariate values. For example, if two hypothetical individuals share the same covariate values but the location random effect for one is 5 units higher than the other individual, then the mean FEV_1 for one will be 5 unit higher than the other.

Similarly, the exponential of the predicted scale random effect captures a subject-specific scaling factor (in terms of "inflation/deflation") of the FEV_1 standard deviation in a population with the same covariate values. For example, if the same two hypothetical individuals share the same covariate values but the scale random effect for one is 0.7 higher than the other, then the FEV_1 standard deviation for one would be $e^{0.7} = 2$ times the standard deviation of the other. The predicted scale random effect value provides the individual component (in addition to the common component determined by the covariate values) of

the within-individual variability.

Prior predictive checks [20] induced a choice of generic informative priors:

- normal prior for linear fixed effects (all centered at 0 except the one for the intercept of the mean submodel, centered at 2)
- Exp(10) for variances of random effects and smoothing terms (which are specified in the model as random effects)
- Lewandowski-Kurowicka-Joe LKJ(2) prior for random effect correlation matrix R.

The specification of the age-sex interaction in the package `brms` [16] is based on the package `mgcv` [21]. The factor-smooth interaction in `mgcv` specifies a different smoothing function for each sex with a centering constraint with respect to the model intercept. Therefore, to account for sex mean differences, a main parametric term for sex is also included. More details are available at https://stat.ethz.ch/R-manual/R-devel/library/mgcv/html/gam.models.html.

B Additional figures

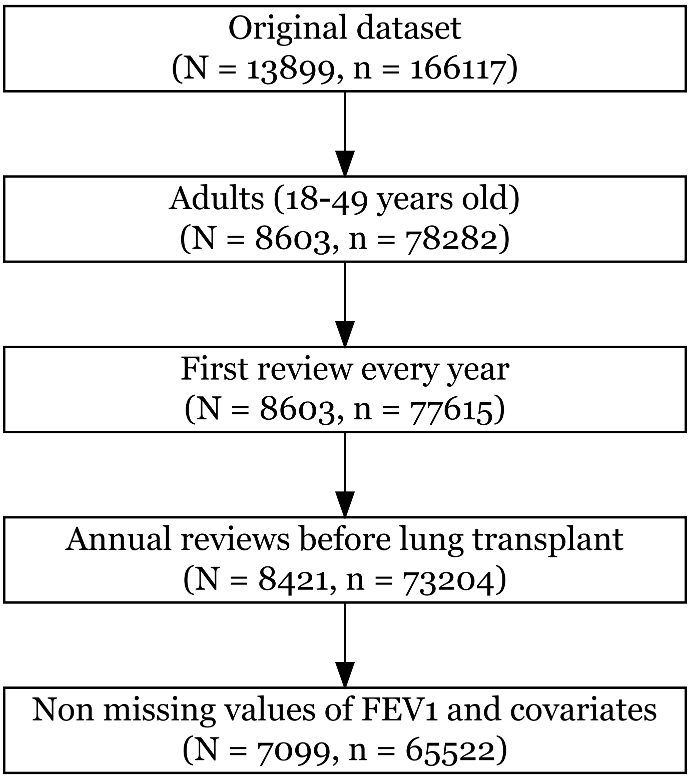

Figure S.1: Diagram of inclusion criteria with number of individuals N and number of observations n.

B.1 Results about interactions, predictions and random effects

The parametric term for sex (accounting for the average difference between male and female smooth functions) and the corresponding 95% credible interval is equal to 0.10 (0.08; 0.12) in the mean submodel

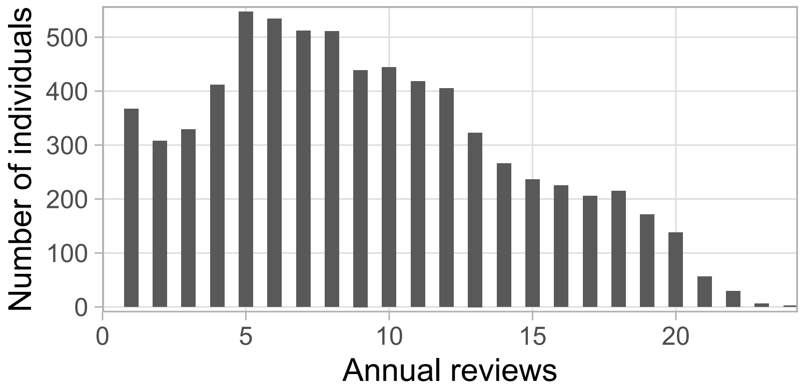

Figure S.2: Histogram of individuals by number of annual reviews.

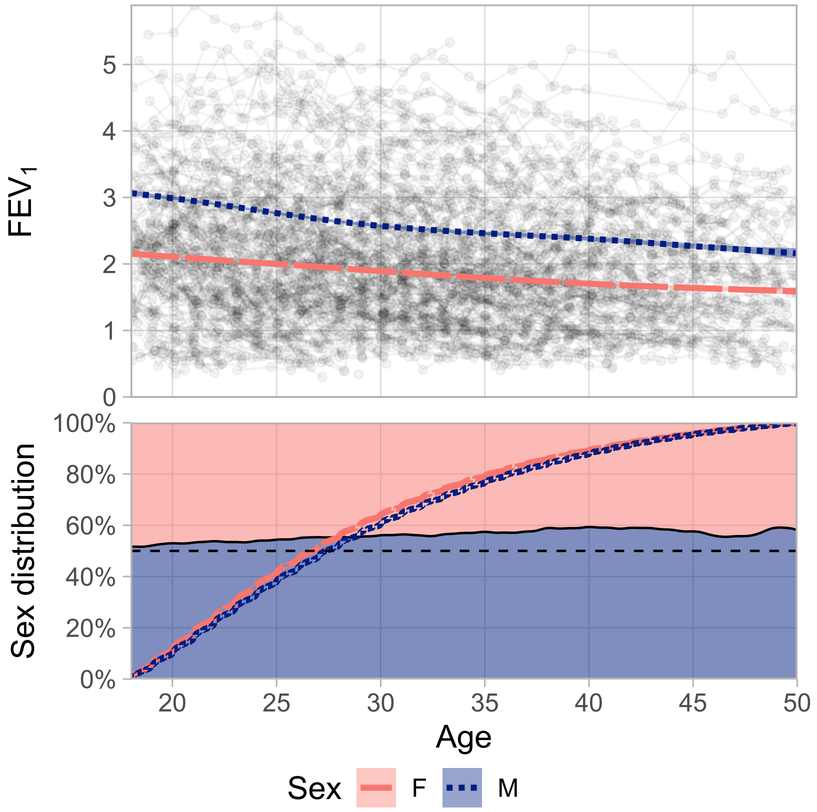

Figure S.3: Top: Spaghetti plot for raw FEV_1 vs. age. Each black line connects the dots representing the lung function measurements for each individual at each annual review. The smoothed lines depicts the trend for females (red) and males (blue) with corresponding confidence intervals. For plotting clarity, only a random subset of dots and lines is reported.
Bottom: Sex distribution in the dataset selected for the analysis. The red and blue lines show the empirical cumulative distribution function over age for females (red) and males (blue), giving at each age level a the percentage of individuals younger than a. The black solid line shows the relative percentage of males and females at each age point. The black dashed line marks the 50% level.

and 0.25 (0.22; 0.27) in the variability submodel. These values can be observed as the average difference between the lines displayed in Figure 1.The value of 0.10 liters for the average sex difference is not dissimilar from the value of 0.08, that is the difference between the age main effects for males and females in the GLI equations (see 22 and lookup tables in `https://www.ers-education.org/lr/show-details/?idP=138978`).

Given these parameter estimates, we can predict the FEV_1 mean and standard deviation trajectories for specific covariate values. Some results are shown in Figure S.4.

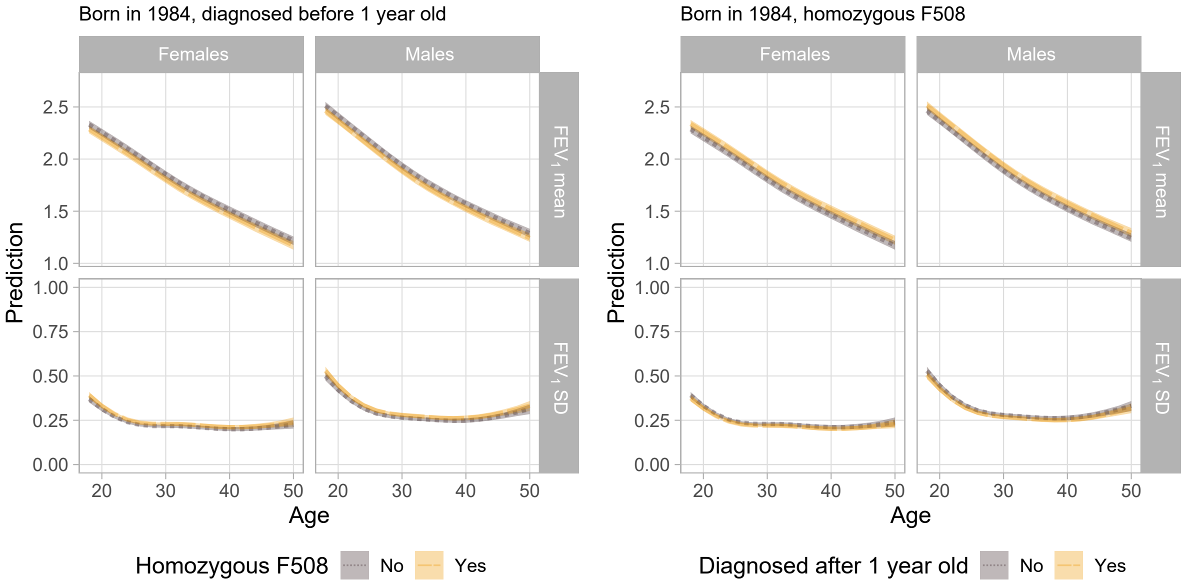

Figure S.4: Posterior predictions of mean and standard deviation of FEV_1 by sex and age for specific sets of covariate values. Year 1984 is the median year of birth in the population.

The parameters of the distribution of the random effect matrix are also estimated (Table 2). For the mean submodel, the standard deviation of the random intercept is equal to 1.02. This means that if we consider a normal distribution with mean zero and this standard deviation, 95% of the individuals will get a predicted random effect in the range of (plus or minus) $1.96 \cdot 1.04 = 2.04$ FEV_1 litres. The posterior mean of the standard deviation of the scale random effect is equal to 0.45 and most of the posterior density lies far from the lower bound of 0 (see Figure S.5 in the Supplementary Material for the full posterior distribution), indicating that there is some heterogeneity that is not captured by the covariates in the model.

Looking at the individual estimates of the random effects jointly for location and scale (Figure S.6), the point cloud is centered around 0 as per the model specification (statistical model described in Equation (3)). The slope of the least squares regression of the location random effects onto the scale random effects is positive and the sign agrees with the correlation parameter in Table 2.

The relationship between scale random effect estimates and sample size is displayed in Figure S.7. A noteworthy result is obtained for individuals with low number of annual reviews, for which the scale

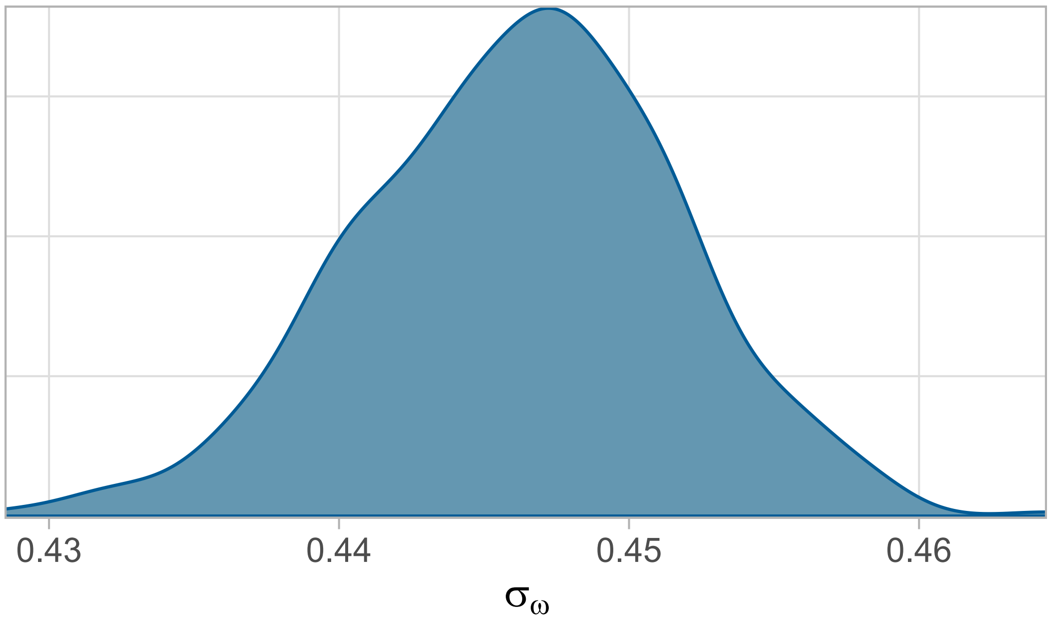

Figure S.5: Posterior density of the standard deviation of the scale random effect σ_ω.

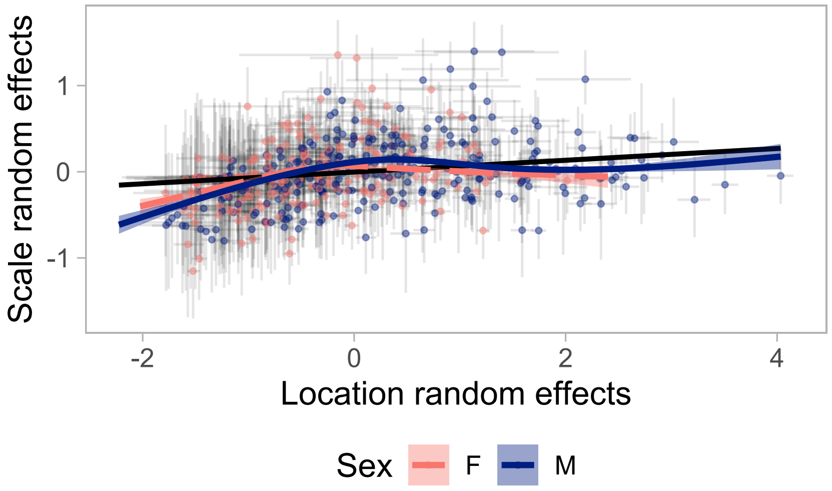

Figure S.6: Plot of the relationship between location and scale random effects. Each individual is represented by a dot and a cross. The dot represents the point estimates for the individual location and scale random effects. The horizontal line of the cross refers to the 95% posterior interval for the location random effect, while the vertical line indicates the same interval for the scale random effects. The black line is the OLS fit for the regression of the location random effect onto the scale random effects, while the blue and red line give the cubic regression spline trend for males and females, respectively. For plotting clarity, only a subset of dots and crosses is reported.

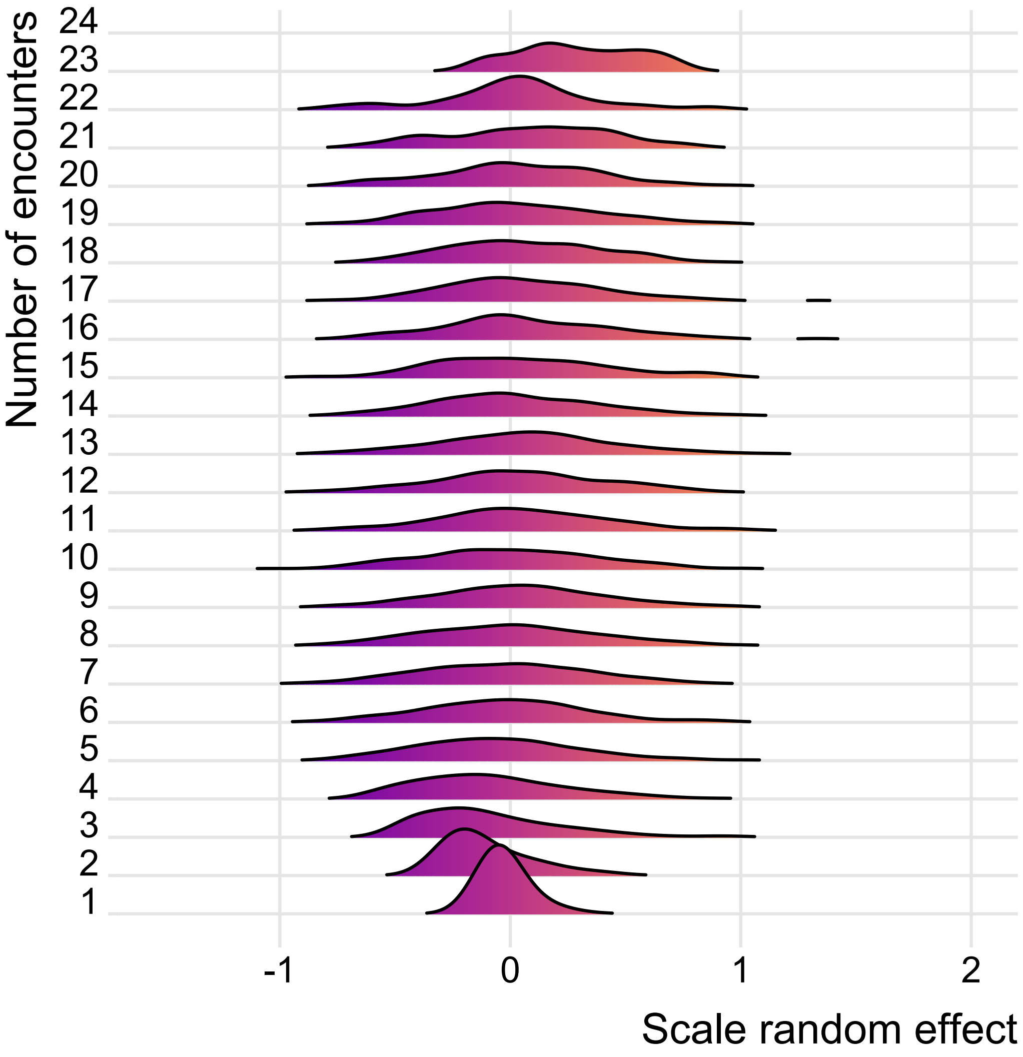

Figure S.7: Distributions of scale random effects grouped by number of annual reviews per individual.

random effects remain closer to zero. The model picks more information about the subject-specific deviation from common (fixed-effects) variability when the number of reviews increases.

B.2 Model diagnostics

The Gelman-Rubin ratio convergence diagnostic for all the parameters ranged between 1 and 1.05, indicating that convergence has been reached. As a graphical assessment of quality of fit, the simulated posterior densities are plotted against the observed FEV_1 distribution (Figure S.8). This procedure is an example of a posterior predictive check [20], that aims at evaluating in a qualitative way whether the model can generate data which resemble the observed data. The overall shape is mostly captured, although some discrepancy occurs in the lower part of the domain.

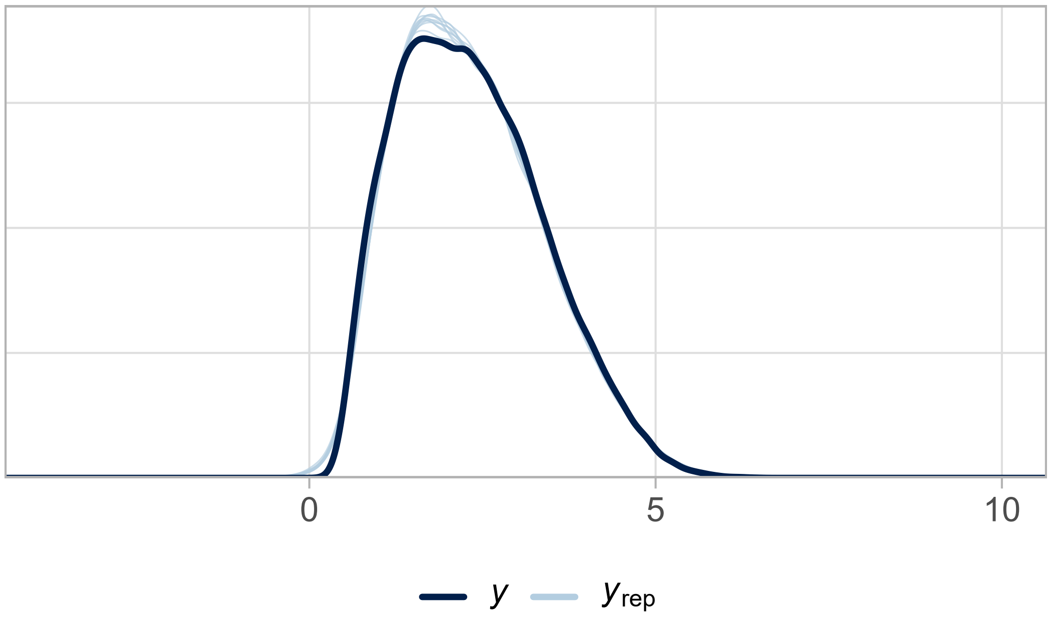

Figure S.8: Posterior predictive checks. The dark blue line represents the density of the outcome (FEV_1) in the dataset, while the lighter lines are 10 simulated posterior distributions generated from the model.

A model with an additional parameter for skewness (i.e. with a skew-normal distribution with the same specification for mean and variance) did not improve the fit (results not reported here).
